# The changing of α5‐GABAA receptors expression and distribution participate in sevoflurane‐induced learning and memory impairment in young mice

**DOI:** 10.1111/cns.14716

**Published:** 2024-05-02

**Authors:** Shengran Wang, Sixuan Wang, Zhun Wang, Jinpeng Dong, Mengxue Zhang, Yongan Wang, Jianyu Wang, Beichen Jia, Yuan Luo, Yiqing Yin

**Affiliations:** ^1^ National Clinical Research Center for Cancer Tianjin Medical University Cancer Institute and Hospital Tianjin China; ^2^ Key Laboratory of Cancer Prevention and Therapy Tianjin China; ^3^ Tianjin's Clinical Research Center for Cancer Tianjin China; ^4^ State Key Laboratory of Toxicology and Medical Countermeasures Beijing Institute of Pharmacology and Toxicology Beijing China; ^5^ Department of Pharmaceutics, School of Pharmacy Shenyang Pharmaceutical University Benxi China

**Keywords:** changing, expression and distribution, neurotoxic mechanism, partial long‐term learning and memory impairment, sevoflurane, α5‐GABAAR

## Abstract

**Background:**

Sevoflurane is a superior agent for maintaining anesthesia during surgical procedures. However, the neurotoxic mechanisms of clinical concentration remain poorly understood. Sevoflurane can interfere with the normal function of neurons and synapses and impair cognitive function by acting on α5‐GABAAR.

**Methods:**

Using MWM test, we evaluated cognitive abilities in mice following 1 h of anesthesia with 2.7%–3% sevoflurane. Based on hippocampal transcriptome analysis, we analyzed the differential genes and IL‐6 24 h post‐anesthesia. Western blot and RT‐PCR were performed to measure the levels of α5‐GABAAR, Radixin, P‐ERM, P‐Radixin, Gephyrin, IL‐6, and ROCK. The spatial distribution and expression of α5‐GABAAR on neuronal somata were analyzed using histological and three‐dimensional imaging techniques.

**Results:**

MWM test indicated that partial long‐term learning and memory impairment. Combining molecular biology and histological analysis, our studies have demonstrated that sevoflurane induces immunosuppression, characterized by reduced IL‐6 expression levels, and that enhanced Radixin dephosphorylation undermines the microstructural stability of α5‐GABAAR, leading to its dissociation from synaptic exterior and resulting in a disordered distribution in α5‐GABAAR expression within neuronal cell bodies. On the synaptic cleft, the expression level of α5‐GABAAR remained unchanged, the spatial distribution became more compact, with an increased fluorescence intensity per voxel. On the extra‐synaptic space, the expression level of α5‐GABAAR decreased within unchanged spatial distribution, accompanied by an increased fluorescence intensity per voxel.

**Conclusion:**

Dysregulated α5‐GABAAR expression and distribution contributes to sevoflurane‐induced partial long‐term learning and memory impairment, which lays the foundation for elucidating the underlying mechanisms in future studies.

## Graphical abstract

The increase in unphosphorylated Radixin causes the diffusion of α5‐GABAARs from the extra‐synaptic space to the synaptic cleft. α5‐GABAARs are localized to a smaller area at synaptic cleft but in unchanged confinement space at extra‐synaptic space, with reduced expression in the extra‐synaptic space but no change in the synaptic cleft, but all resulted in an increase in fluorescence intensity per voxel.

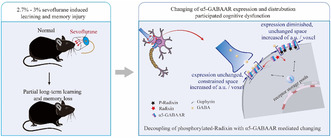

## 1 INTRODUCTION

Sevoflurane is the most commonly used inhaled anesthetic medication, which has a better anesthetic effect and faster metabolism compared to other anesthetic drugs.^1^, ^2^, ^3^, ^4^ In clinical practice, approximately 1–2 Minimum Alveolar Concentration (MAC) of sevoflurane is used to maintain anesthesia during surgery, and toxicological and pharmacological evaluation data confirm this anesthetic concentration as clinically safe.^5^, ^6^, ^7^, ^8^ However, an increasing number of clinical studies have found that the clinical concentration of sevoflurane also has potential neurotoxicity, which can cause cognitive impairment.^2^, ^9^, ^10^ The impairment of learning and memory functions in the brain is closely related to the decrease in synaptic plasticity in the hippocampus.^11^, ^12^ Numerous studies have found that anesthetic drug‐induced cognitive impairment is inseparable from the imbalance of excitatory and inhibitory neural electrical signals in the hippocampus, and this decrease in synaptic plasticity is related to the imbalance of inhibitory neurotransmitter receptor expression on the synaptic surface.^13^, ^14^, ^15^ How these learning‐related synaptic changes occur has not been fully elucidated yet.

α5‐Subunit‐containing GABAA receptors (α5‐GABAARs) are highly expressed in the hippocampus,^16^ primarily located in the extrasynaptic regions, binding with phosphorylated Radixin to mediate tonic inhibition.^16^, ^17^ A small number of α5‐GABAARs located in the synaptic cleft bind to the postsynaptic scaffolding protein gephyrin, mediating phasic inhibition (miniature inhibitory postsynaptic currents, mIPSCs).^18^ These receptors are essential for the sensitivity of body to inhaled anesthetic medication at low concentrations, bidirectionally controlling two types of inhibitory currents, affecting synaptic homeostasis, and resulting in decreased learning and memory capabilities in animals.^18^, ^19^, ^20^, ^21^, ^22^, ^23^ Orser and colleagues have confirmed that an anesthetic maintenance concentration of about 1 MAC easily produces extremely low concentrations of residual anesthetics in brain.^20^, ^22^, ^24^, ^25^, ^26^, ^27^ The residual anesthetics enhance tonic inhibition around the extra‐synaptic space, suppressing the transmission of excitatory signals and subsequently leading to postoperative cognitive decline, which is also known as amnesia.^20^, ^23^ However, the molecular mechanism by which α5‐GABAAR mediates synaptic plasticity changes affecting cognitive function levels is not clear.

Herein, we initially construct an anesthesia model in young mice with a maintained sevoflurane concentration of 2.7%–3.0% (approximately 1MAC),^6^ evaluating its long‐term cognitive ability and analyzing the differential genes, based on animal behavioral experiments and RNA‐Seq technology. Subsequently, we evaluate the expression of α5‐GABAAR in the synapse and its upstream and downstream protein expression levels after anesthesia through protein quantification and morphological methods. Afterwards, hippocampal longitudinal slices were obtained for study, and the spatial distribution of α5‐GABAAR at the neuronal soma sites was analyzed, employing histology and three‐dimensional imaging techniques. This elucidated the changes in the dual anchoring positions of α5‐GABAAR and their association with the Gephyrin and ROCK‐Radixin phosphorylation signaling pathways. This study provides a theoretical contribution to our clarification of the involvement of α5‐GABAAR double anchoring variation in the decline of synaptic plasticity mediating the decrease in learning and memory abilities caused by sevoflurane.

## 2 EXPERIMENTAL METHODS

### 2.1 Animal experiments

All told, 40 female C57/BL6 mice (aged 3 months) were used in this research, and bought from the SPF Biotechnology Co., Ltd. (Beijing, China). All animal experiments involved in the following study were executed according to the US National Institute of Health (NIH) Guide for the Care and Use of Laboratory Animals published by the US National Academy of Sciences, and approved by the Animal Ethical and Welfare Committee of Tianjin Medical University Cancer Institute and Hospital, Tianjin, China.

### 2.2 Establishment of sevoflurane exposure model

The model of learning and memory impairment induced by sevoflurane was prepared as previously described.^19^

For behavioral analyses, the Morris water maze (MWM, Beijing Shuoji Technology Co., Ltd., Beijing, China) was employed to evaluate the learning and memory capabilities of the mice. The MWM is a reliable tool for assessing learning and memory in mice, comprising a round swimming pool (diameter, 122 cm; depth, 50 cm) and a platform (0.5–1 cm beneath the surface) submerged in lightproof water (warmed at 22 ± 2°C).

The basic procedures for the MWM test were conducted according to *nature protocol*, which includes a place navigation trial and a spatial probe trial, briefly described below.^28^ After a 7–14 days adjustment period, the mice with normal cognitive function were randomly divided into two groups (*n* = 7 per group: young mice sevoflurane exposure group (SEVO), young mice control group (CON)). Next, the SEVO mice were placed in a transparent container for an hour and deeply anesthetized with 2.7%–3% sevoflurane delivered in 2 L/min oxygen. Then 1 day later, mice were repeatedly placed in the maze at a new start virtual quadrant (four virtual quadrants: I, II, III, and IV), trained to arrive the circular escape platform in the second quadrant. During the navigation trial, each mouse experienced 4 trials per day for 5 consecutive days. In the spatial probe trial, the escape platform was removed, and the mice were placed in the opposite location (quadrant III) to the target quadrant (quadrant II). Each mouse was given 60 s to search for the platform. The swimming trajectory and data of the mice were automatically captured by a camera with SLY‐WMS Morris analysis system.

### 2.3 The α5‐GABAAR and relative proteins quantitative analysis

Twenty‐four hours after anesthesia, the Western blot method was used to analyze the expression levels of α5‐GABAAR, Gephyrin, Radixin, P‐ERM (Phospho‐Ezrin [Thr567]/Radixin [Thr564]/Moesin [Thr558]), P‐Radixin (Phospho‐Radixin [Thr564]), ROCK, and IL‐6.

Semi‐quantitative analysis (Western blot): hippocampi were isolated from the SEVO and the CON as previously described. α5‐GABAARs from hippocampi were split by electrophoresis and then transferred onto polyvinylidene fluoride membranes. Primary antibodies against α5‐GABAAR, Gephyrin, Radixin, P‐ERM, P‐Radixin, ROCK, IL‐6, Actin (Abcam, Cambridge, MA) were used, followed by the appropriate HRP‐conjugated secondary antibody (Abcam, Cambridge, MA). Finally, the membranes were exposed to lighted with Ultra ECL WB Substrate kit (Invigentech, CA, USA).

Additionally, to objectively evaluate the changes in α5‐GABAAR, Gephyrin, Radixin, ROCK1, and ROCK2, Real‐time quantitative PCR (RT‐PCR) was performed using LightCycler® 480 II Real‐time PCR Instrument (Roche, Switzerland) with 10 μL PCR reaction mixture comprising 1 μL of cDNA, 5 μL of 2 × PerfectStart™ Green qPCR SuperMix, 0.2 μL each of forward and reverse primers, and 3.6 μL of nuclease‐free water. Reactions were incubated in a 384‐well optical plate (Roche, Switzerland) at 94°C for 30 s, followed by 45 cycles of 94°C for 5 s and 60°C for 30 s. Each sample was analyzed in triplicate.

Fluorescence images of longitudinal hippocampus slices (8 μm thickness) were captured using Pannoramic Scan and visualized using Case Viewer 2.4 (3DHISTECH Ltd.).

### 2.4 Immunolabeling and three‐dimensional (3D) spatial distribution analysis of α5‐GABAAR

Twenty‐four hours after anesthesia, the expression and distribution analysis of α5‐GABAAR, Gephyrin, P‐ERM, and SV2 were supported by experiments involving immunofluorescence (IF) staining.

The process for IF staining of the longitudinal section of the hippocampus is as follows: the soma of hippocampal neurons was stained with primary antibodies (anti‐α5‐GABAAR, anti‐SV2), followed by application of corresponding fluorescence‐conjugated secondary antibodies for 2 h at room temperature. Images of all samples were captured using an upright confocal microscope (Zeiss LSM 980), and then rendered and recorded using the surface and snapshot function in Imaris v9.8. The brightness of images was equalized utilizing Photoshop 2021 (Adobe).

The surface rendering of α5‐GABAAR and SV2 was conducted using Imaris v9.8 software. The surface‐surface colocalization analysis of α5‐GABAAR and SV2 were performed, and the voxel total number counts of α5‐GABAAR, SV2, and their overlapping areas were analyzed using Imaris Measurement Pro software and ImarisColoc software.

### 2.5 Transcriptome analysis

The transcriptome analyses were executed to assess alterations in total mRNA following young mice 24 h post‐anesthesia.

The protocol for transcriptome analysis was given from OE Biotechnology Co., Ltd. (Shanghai, China) (https://www.oebiotech.com/). The main workflow is as follows: total RNA was extracted using the mirVanaTM RNA Isolation Kits (ThermoFisher) according to operating manual, quantified by the NanoDrop ND‐2000 (ThermoScientific), and its integrity was evaluated using Agilent Bioanalyzer 2100 (Agilent Technologies). After a series of experimental process, Cyanine‐3‐CTP (Cy3) labeling cRNA was transferred and purified from RNA and hybridized with Chip (Agilent). Then this chip was scanned using Agilent Scanner G2505C (Agilent Technologies), and the results of one‐color microarray data normalized with Feature Extraction Software (Agilent).

For statistical analysis, the original data were obtained by the Feature Extraction software (version10.7.1.1, Agilent Technologies) from array images. Subsequently, these statistical data were normalized with the quantile algorithm. The probes detected with at least 75.0% in 6 samples were decided for following data analysis. The fold change and *p* calculated with *t*‐test were used to identify up‐ and down‐regulated genes, which had corresponding thresholds (a fold change) ≥2.0 and *p* ≤ 0.05. First, the unsupervised‐learning principal component analysis (PCA) was conducted in R (v4.0) using standard prcomp function for identifying the differences after anesthesia, and then RNAs were clustered into two groups based on differential genes by R‘pheatmap’ package with the parameter of method = “ward. D.” Subsequently, these differentially expressed mRNAs were determined their roles by gene ontology (GO) and KEGG pathways (Release 92.0) working by R ‘cluster Profiler’ (4.0) package, and we delimit *p* < 0.05 as effective enriched. Finally, Hierarchical Clustering was performed to display the differential gene expression patterns between the two groups.

### 2.6 Statistical analysis

In this study, quantitative results were derived from a minimum of three specimens and three independent experiments, each represented as mean ± standard error of mean (SEM). In this study, Shapiro–Wilk test for normality were used to assess data distribution. Unpaired Student's *t*‐test was used to compare differences between SEVO and CON, when the data conform to a normal distribution. Otherwise, Mann–Whitney test was used to analyze data that do not exhibit a normal distribution between SEVO and CON. In addition, a two‐way ANOVA, Šídák's multiple comparisons test, is used to estimate the results of behavioral experiments in space learning. All data analyses were performed using GraphPad Prism 9.5.0 (GraphPad, San Diego, CA), with *p* values represented as follows: *p < 0.05, **p < 0.01, ***p < 0.001, ^#^ *p* < 0.05, and ^##^ *p* < 0.01.

## 3 RESULTS

### 3.1 Partial long‐term learning and memory impairment in mice following sevoflurane exposure

**FIGURE 1:**
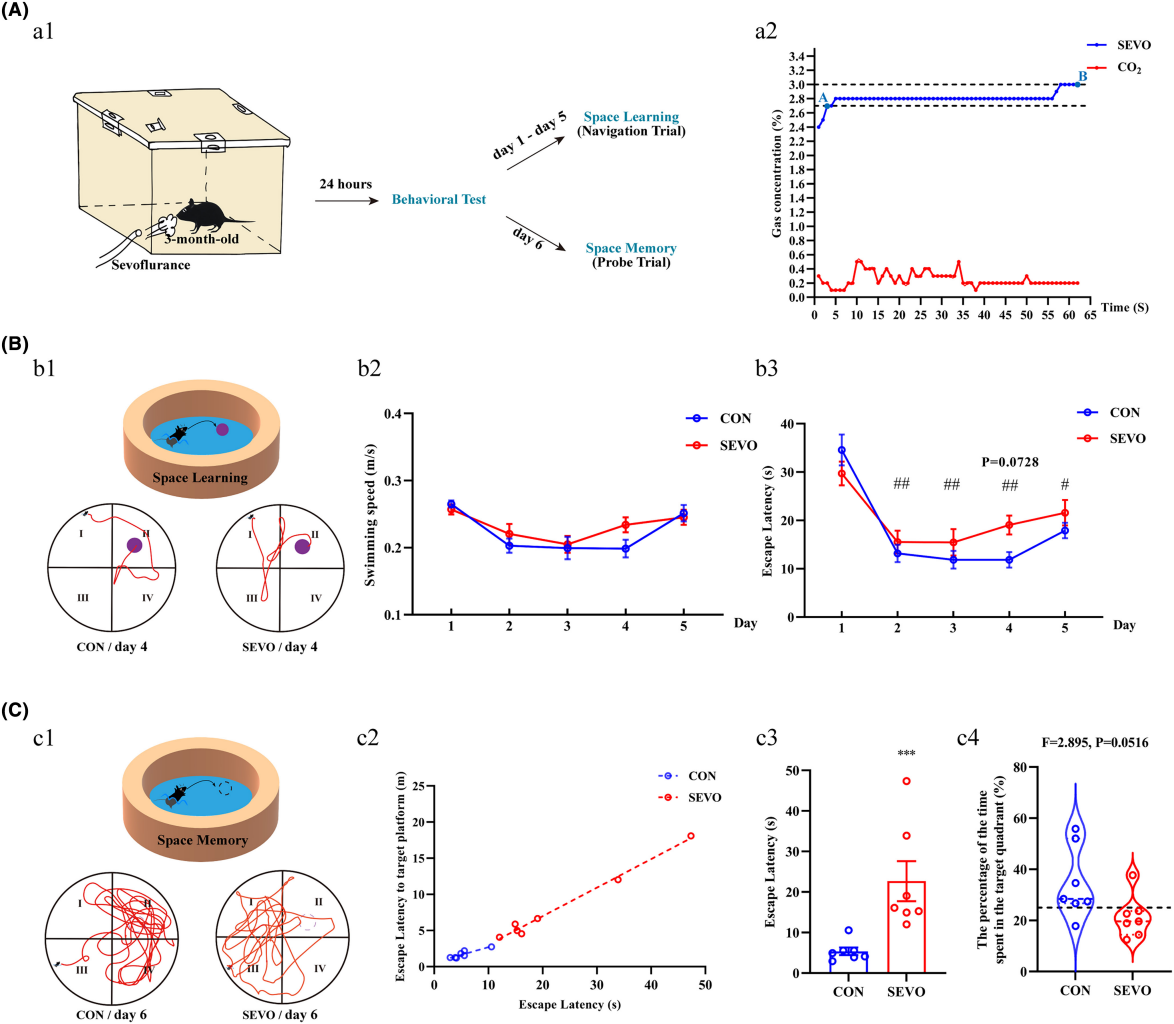
The evaluation of learning and memory after sevoflurane exposure. (A) Diagrammatic presentation of the mainly procedures leading to the fabrication and identification of learning and memory (a1), and anesthesia time and concentration (a2). (B) Swimming trajectory of mice on the fourth day post‐sevoflurane exposure in SEVO, and CON as Control (b1). Line chart comparing the swimming speed and escape latency of reaching the platform (*n* = 7) (b2, b3). (C) Swimming trajectory of mice on the sixth day in SEVO and CON (c1). Scatter diagram and histogram comparing of distance and time to first cross the virtual platform (*n* = 7) (c2, c3), and violin plot showing the percentage of time in target quadrant (*n* = 7) (c4). ***p < 0.001 versus CON. *p* = 0.0728 versus CON on the fourth day. In the CON group: ^#^ *p* < 0.05, ^##^ *p* < 0.01 versus CON on the first day.

The behavioral test of MWM was utilized to evaluate the learning and memory capabilities post‐anesthesia, as depicted in the procedural diagram (Figure 1A:a1). Following a 1‐h anesthesia with 2.7%–3% sevoflurane (Figure 1A:a2), mice in the SEVO group required approximately 19 s (mean time = 19.0532 s) to reach the platform, whereas those in the CON group took about 12 s (mean time = 11.8465 s), despite the lack of a statistically significant difference between the groups (Figure 1B:b1–b3). After training 5 days, the anesthetized mice spent more time (Figure 1C:c3), although did not cover a greater time in their search for the virtual platform in the target quadrant (Figure 1C:c4). Overall, the mice in SEVO minimized the distance and time to target platform (Figure 1C:c1, c2). These results indicate that partial long‐term spatial learning and memory abilities of the mice were injured after anesthesia exposure.

### 3.2 Immunosuppression after sevoflurane exposure

**FIGURE 2 2:**
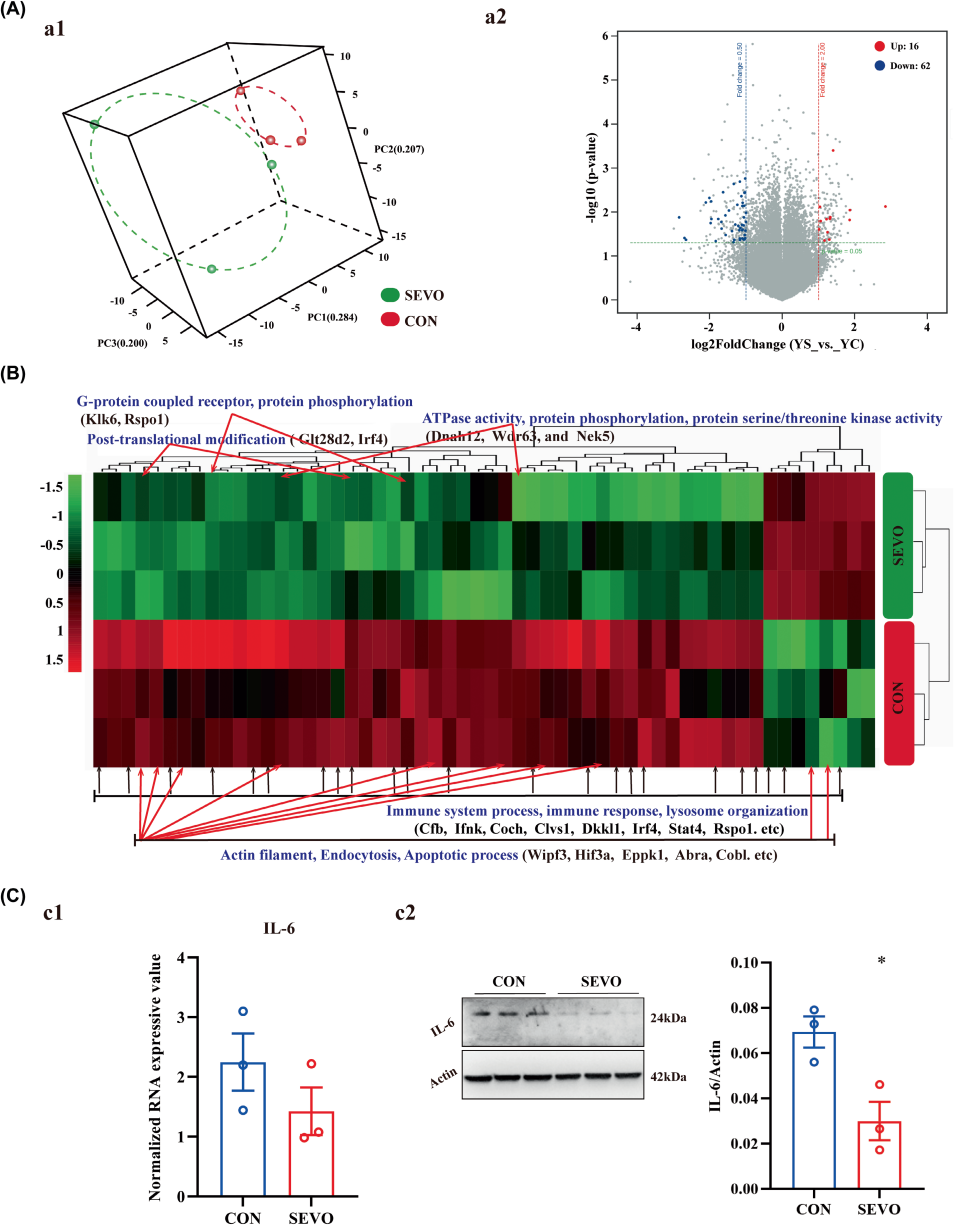
Change of hippocampal transcriptome after anesthesia. (A) 3D principal component analysis showing differences in total mRNA in whole‐hippocampus were analyzed using gene chip method (*n* = 3) (a1); The differential expressed genes were illustrated using volcano plot (*n* = 3) (a2). (B) Heatmap of the mRNAs was normalized with the quantile algorithm between CON and SEVO. Significantly enriched downregulated (green) and upregulated (red) GO biological processes (|Fold Change| ≥ 2; *p* ≤ 0.05). Scale bar explains level of expression (red represents upregulated, green represents downregulated) (*n* = 3). (C) Histograms showing Normalized RNA expressive value of IL‐6 using gene expression microarray (c1); Histograms show semi‐quantitative analyses for IL‐6 protein conducted by Western blot (*n* = 3) (c2). *p < 0.05 versus CON.

To seek the underlying molecular mechanism, the hippocampal transcriptome was analyzed and a total of 56746 RNAs were probed by Agilent SurePrint G3 Mouse Gene Expression v2 8 × 60K Microarray (DesignID: 074809). The distribution of all samples showed a distinction between CON and SEVO (Figure 2A:a1), and 16 genes were up‐regulated and 62 genes were down‐regulated (Figure 2A:a2). The reductions were related to the learning and memory of immune response, protein phosphorylation, ATPase activity, and actin filament binding (Figure 2B). The downregulation of IL‐6 was evaluated by western blot (Figure 2C:c2), although IL‐6 unchanged according to gene chip (Figure 2C:c1).

### 3.3 The expression perturbations of α5‐GABAAR in hippocampus

**FIGURE 3:**
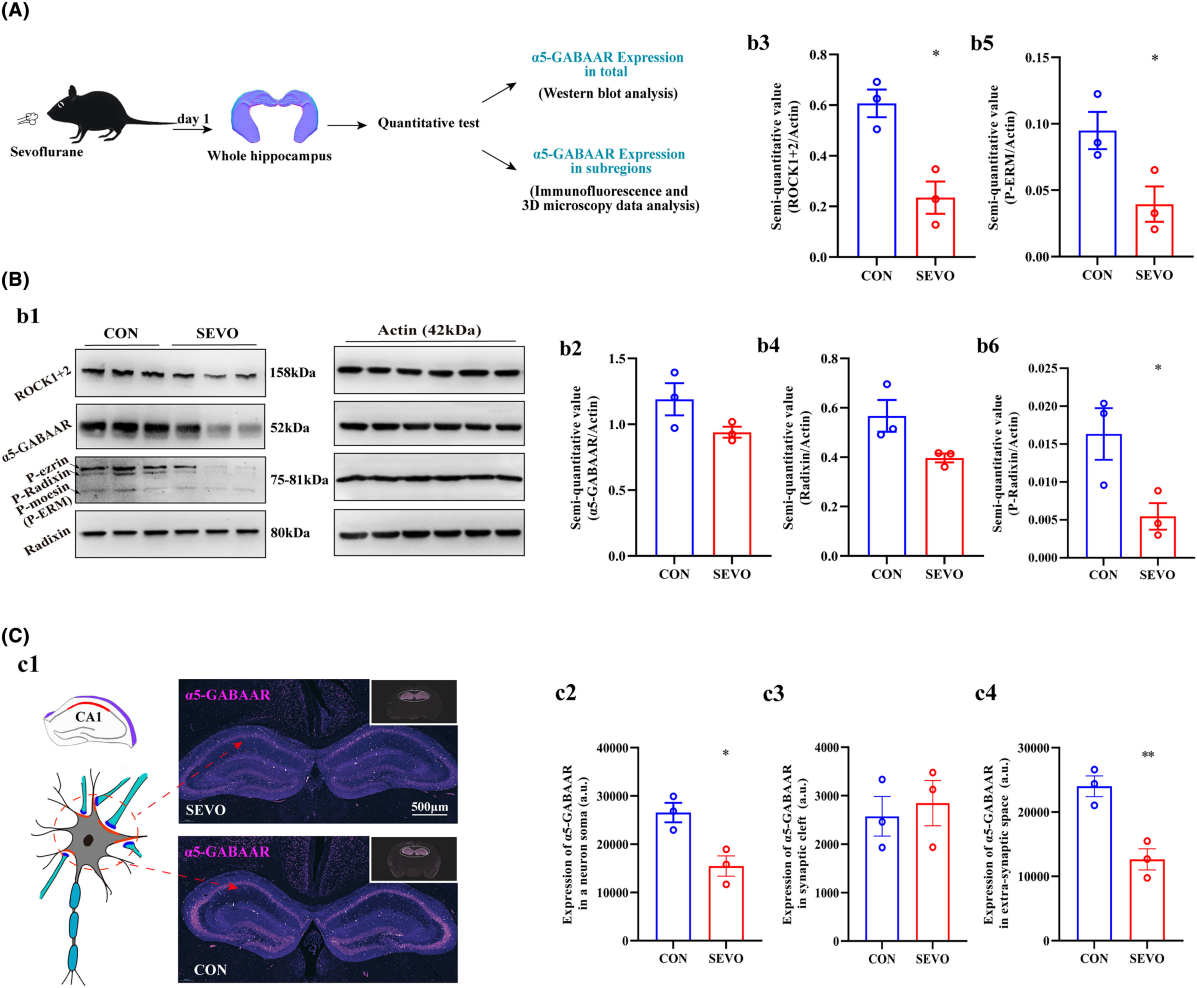
The expression of α5‐GABAAR after anesthesia in hippocampus. (A) Schematic showing the expression of α5‐GABAAR in whole‐hippocampus were analyzed using Western blot and IF (*n* = 3). (B) Results from Western blot, along with a chart illustrating the expression of α5‐GABAAR, Radixin, P‐ERM, P‐Radixin, and ROCK in the whole hippocampus (*n* = 3) (b1–b6). (C) The coronal plane image of the hippocampus, immunostained for α5‐GABAAR (pink) and processed using Adobe Illustrator, is shown in c1. The location of neuronal somata in the CA1 area is also illustrated using cartoons (c1). Scale bar, 500 μm. The bar chart showing variation of α5‐GABAAR expression among CA1 subregion in hippocampus (c2–c4) (*n* = 3). *p < 0.05, **p < 0.01 versus CON. Unit of fluorescence intensity = arbitrary units (a.u.).

To investigate the expression level of α5‐GABAAR in whole hippocampus and subregions (CA1, CA2, CA3, and DG). Three‐month‐old mice was anesthetized and sacrificed, and whole hippocampus was operated as exhibited in the graph (Figure 3A). We first evaluate the expression of α5‐GABAAR in whole region of hippocampus between CON and SEVO. The α5‐GABAAR expression level unchanged according to the results of western blotting and RT‐PCR (Figure 3B:b1,b2 and Figure S1D). In addition, hippocampal coronal plane (8 μm thickness) was labeled with α5‐GABAAR using immunofluorescence, and the majority of α5‐GABAARs are located in the neuronal soma (Figure 3C:c1). The level of α5‐GABAAR expression varied across the right and left hippocampus and the three subregions CA2, CA3, and DG. Basically, the expression of α5‐GABAAR decreased in the extra‐synaptic space and whole neuron soma, as well as in the right and left hippocampus in each subregion (Figures S2A–C and S3A–C), but unchanged in the synaptic cleft (Figure S4A–C). In addition, hippocampus CA1 is closely related to spatial learning and memory. The lower expression of α5‐GABAAR in CA1 whole neuronal somata and extra‐synaptic space after sevoflurane treated (Figure 3C:c2,c4), but we have not detected expression changes of it in synaptic cleft (Figure 3C:c3). The expression level of the anchoring protein P‐Radixin, which is associated with α5‐GABAAR in the synaptic membrane, is a critical factor influencing the expression and distribution of α5‐GABAAR between the synaptic cleft and the extra‐synaptic space. The expression levels of Radixin, P‐ERM, P‐Radixin, and ROCK were primarily quantified using Western blot (Figure 3B:b1), and Radixin and ROCK were also quantified using RT‐PCR (Figure S1A–C). The charts indicate that Radixin expression was nonspecific (Figure 3B:b4), whereas the quantitative analyses revealed decreased levels of P‐ERM, P‐Radixin, and ROCK (Figure 3B:b3,b5,b6).

### 3.4 The confinement space and fluorescence intensity of α5‐GABAAR in synaptic cleft and extra‐synaptic membrane

**FIGURE 4:**
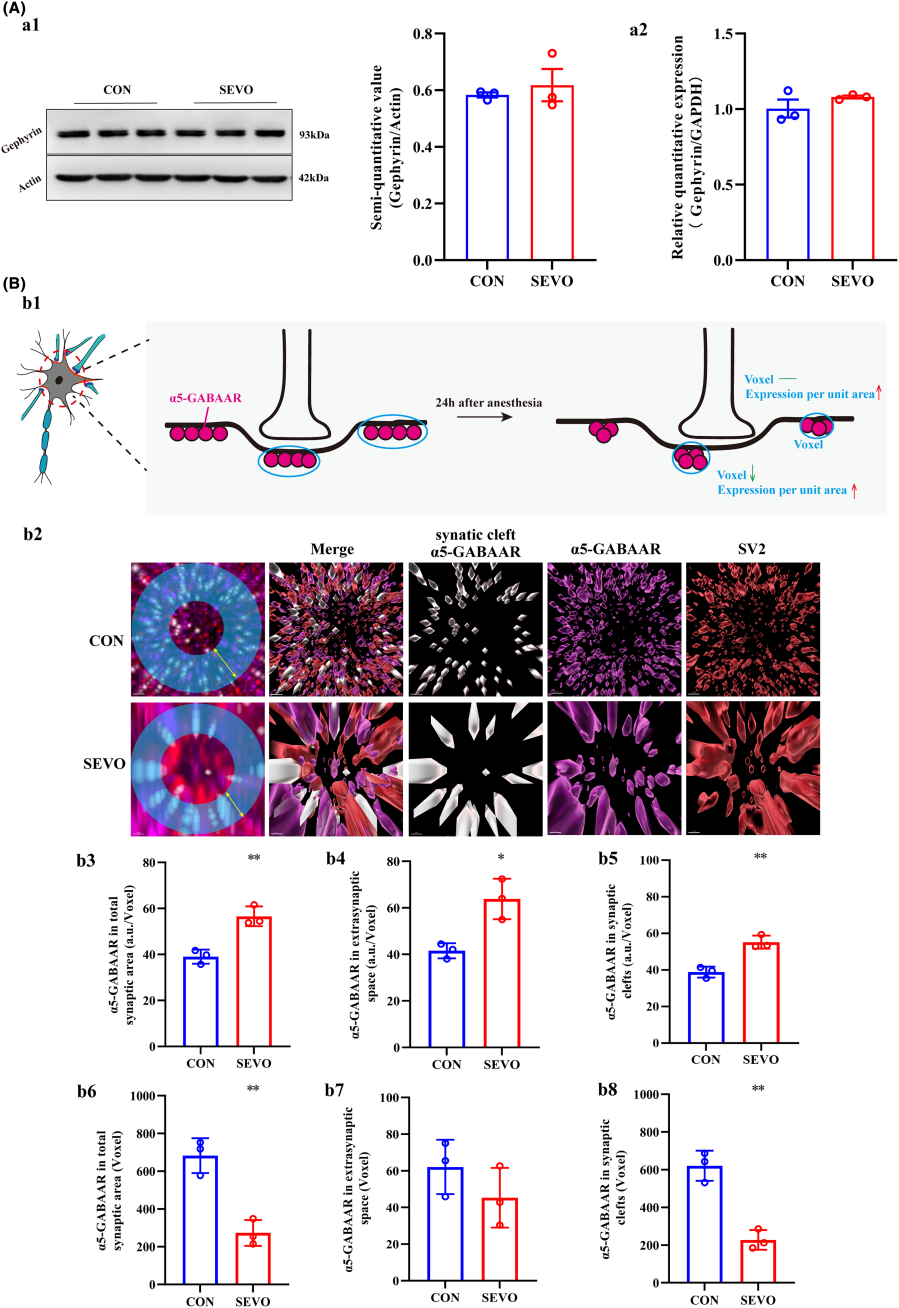
The spatial variation of α5‐GABAAR in synaptic cleft and extra‐synaptic membrane. (A) Anchoring proteins, gephyrin, were quantified using RT‐PCR and Western blot (a1, a2) (*n* = 3). (B) The space confinement size of synaptic α5‐GABAAR is displayed using 3D images from cartoon and immunostaining (b1, b2). Column graphs exhibit the degree α5‐GABAAR aggregation after sevoflurane treated. Space confinement size of α5‐GABAAR is displayed using voxel (b6–b8), and fluorescence intensity of α5‐GABAARs per unit area in CA1 synaptic space and extra‐synaptic space in 30 neuronal somata (b3–b5). *p < 0.05, **p < 0.01 versus CON. Unit of fluorescence intensity = arbitrary units (a.u.).

As evident from the results above, cartoon shown that in CA1 the expression of α5‐GABAAR decreased in extra‐synaptic space, and unchanged in synaptic cleft (Figure 4B:b1). The mRNA and protein expression levels of the anchoring protein gephyrin remained unchanged, which corresponds to the alterations in α5‐GABAAR levels within the synaptic cleft (Figure 4A:a1,a2). But histograms reveal a reduction in the confinement size of α5‐GABAARs within the CA1 synaptic and total synaptic spaces, although unchanged within CA1 extra‐synaptic space (Figure 4B:b6–b8). In addition, an enhancement of the fluorescence intensity of α5‐GABAARs per unit area in the CA1 synaptic space and extra‐synaptic space (Figure 4B:b3–b5). The visual data depicted changes in the distribution of α5‐GABAARs within the neuronal bodies of the annular blue (Figure 4B:b2).

## 4 DISCUSSION

Numerous studies have revealed that the cognitive decline induced by sevoflurane is closely related to its neurotoxicity, manifesting a complex and diverse toxicological and pathological mechanism.^29^, ^30^, ^31^ α5‐GABAAR possesses a unique dual‐anchor structure, primarily distributed both within and surrounding the inhibitory postsynaptic cleft, serving as the main trigger site for cognitive dysfunction and the key synaptic target for sevoflurane.^18^, ^25^, ^32^, ^33^ The molecular mechanism of α5‐GABAAR in learning and memory damage under the clinical concentration of sevoflurane remains to be fully clarified. Our research results indicate that 24 h following 1 h anesthesia treatment with 2.7% to 3% sevoflurane, the ability of synaptic remodeling decreases, and the expression and distribution of α5‐GABAAR on the membrane fail to recover to the normal dual‐anchor level. In the subsequent 5–6 days, the mice exhibit impaired partial long‐term sequential learning and memory abilities, a condition known as anterograde amnesia. There have been reports that the number of α5‐GABAAR, a major variable in regulating synaptic strength, is crucial to the process of memory and cognition. Beverley A Orser et al. (2012) discovered that in genetically mutated animal models lacking α5‐GABAAR, neither isoflurane nor sevoflurane could induce long‐term memory impairment.^25^ Kwakowsky et al. (2018) reported that the upregulation of α5‐GABAARs in the dentate gyrus leads to enhanced tonic inhibition and impaired spatial memory.^34^ It has been validated that the knockout or interference of α5‐GABAARs enhances the cognitive capabilities of experimental animals.^35^, ^36^, ^37^ However, some preclinical studies also have found that blocking α5‐GABAARs may lead to the emergence of behaviors related to schizophrenia, which manifest as cognitive deficits.^38^, ^39^ On the other hand, Soh and Lynch; Rissman; Armstrong et al. reported that compared with normal levels, patients with Alzheimer's disease with low expression of α5‐GABAAR have more severe memory and cognitive decline.^40^, ^41^, ^42^ Our research results show that the expression of α5‐GABAARs on the outer side of the synaptic gap is significantly downregulated in neuronal somata, although the expression of the receptor inside the gap is not affected. Therefore, our research suggests that the downregulation of the number of α5‐GABAARs outside the synapses may be an important factor in the molecular mechanism of learning and memory decline mediated by the disorder of α5‐GABAAR dual‐anchor.

The a5‐GABAAR is enriched in the actin cytoskeleton outside the synaptic cleft through phosphorylated Radixin (Rdx), participating in the generation of tonic currents, and is enriched inside the synaptic cleft through gephyrin, participating in the production of mIPSCs. Dephosphorylation of Radixin could lead to the migration of a5‐GABAAR in the extra‐synaptic space, thereby enriching the number of receptors in the synaptic cleft and potentially disrupting the stability of inhibitory currents leading to cognitive dysfunction.^43^ The spatial distribution and relative density changes of this protein in local regions of neurons are important reflections of the response to environmental changes and play a crucial role in regulating neural plasticity. Influenced by changes in anchoring proteins such as Gephyrin, GABAAR tends to form clusters of varying densities due to site‐specific changes at synapses.^43^, ^44^, ^45^ However, changes in the distribution density of α5‐GABAAR caused by sevoflurane have not been reported yet. This study found that the expression of α5‐GABAAR in the outer side of the synaptic cleft decreases, and its spatial distribution density increases; while the expression of α5‐GABAAR in the inner side of the synaptic cleft remains unchanged, but its spatial distribution density increases. In summary, our study suggests that the abnormal spatial distribution of α5‐GABAAR at dual anchoring sites may be involved in the neurotoxicity caused by sevoflurane.

Numerous pro‐inflammatory factors and their receptors, such as IL‐1β and IL‐6, exist in the hippocampus.^46^, ^47^ Under normal physiological conditions, a balance between pro‐inflammatory factors and anti‐inflammatory factors is maintained, which is crucial for memory and learning processes.^48^, ^49^, ^50^ Research has proven that an excess of inflammatory factors can lead to a reduction in synaptic plasticity and hippocampal neuron apoptosis, subsequently causing cognitive impairment. However, the conclusions on the effect of inflammatory factors on memory are inconsistent. David A. Morilak et al. (2014) found that inhibiting the activity of IL‐6 in rat brains negatively affects their reversal learning abilities.^51^ Studies by Hee‐Pyoung Park et al. (2017) and Zhang Y et al. (2011) found that sevoflurane can reversibly inhibit inflammatory factors, including IL‐6.^52^, ^53^, ^54^ Although, xiangdong Chen, et al. (2022) reported that in cognitive‐impaired rats inhaling sevoflurane, there is an upregulation of neuroinflammation (IL‐1β, IL‐6, and TNF‐α) in the hippocampus.^55^ The effect of sevoflurane on the expression of inflammatory factors may be related to the concentration and duration of anesthesia, as well as organ specificity.^56^ This study found that in the hippocampus of mice with cognitive impairment caused by sevoflurane, the expression levels of IL‐6 decreased, along with a reduction in the phosphorylation level of ERM proteins and a slowing down of the protein phosphorylation process. Min‐Liang Kuo, et al., (2007) and Antony W. Braithwaite, et al., (2018) found that IL‐6 can activate the RhoA/ROCK signaling pathway and RhoA expression, which positively correlated with IL‐6.^57^, ^58^ However, despite the downward trend in ROCK expression and a decrease in energy metabolism, data on how the Rho‐GTPase‐ROCK pathway participates in Radixin phosphorylation and mediates cognitive impairment caused by sevoflurane is still insufficient.

The α5‐GABAAR primarily regulates synaptic plasticity through membrane expression levels, lateral diffusion, and endocytic recycling mechanisms.^59^ Its precise subcellular localization changes with variations in internal and external environmental factors. However, this study has a second limitation as it lacks real‐time receptor transport data of α5‐GABAAR to support lateral diffusion and internalization processes. This is mainly due to inadequate in vivo monitoring technology and the easy quenching of ex vivo subcellular markers, among other reasons.

## 5 CONCLUSION

**FIGURE 5:**
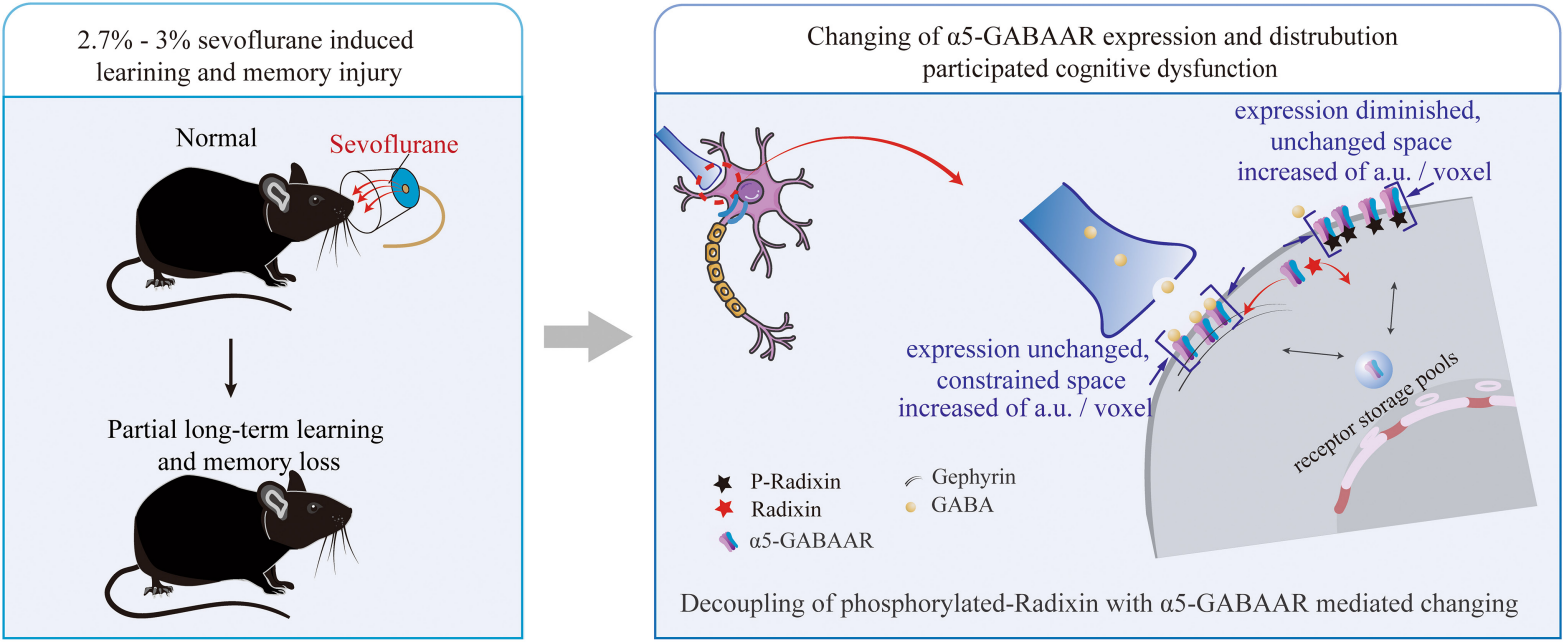
Principal mechanism for α5‐GABAAR participated in sevoflurane‐induced learning and memory impairment. An anesthesia model in young mice with a maintained sevoflurane concentration of 2.7%–3.0% (left). Increasing dephosphorylation of Radixin, activating α5‐GABAAR in extra‐synaptic space partially diffusing into synaptic cleft, where α5‐GABAARs anchored in a smaller confinement size and expressed stronger fluorescence intensity per voxel, although α5‐GABAARs also expressed stronger fluorescence intensity per voxel but stayed in unchanged confinement size in extra‐synaptic space. Interestingly, dephosphorylation of Radixin did not change α5‐GABAARs expression level in synaptic cleft. So wrong anchoring of α5‐GABAARs in synaptic and extra‐synaptic space participate in sevoflurane‐induced learning and memory impairment (right).

Broadly, the increasing dephosphorylation of Radixin has disrupted the distribution and expression of α5‐GABAAR on the membrane following 1 h of anesthesia with 2.7%–3% sevoflurane. On the synaptic cleft, the expression level of α5‐GABAAR did not change, but its spatial distribution was compressed; on the extra‐synaptic space, the expression level of α5‐GABAAR decreased, although its spatial distribution unchanged. This has caused the decline in learning and memory function induced by 2.7%–3% of single sevoflurane anesthesia (Figure 5). Moreover, sevoflurane may inhibit the immune response, causing the immune factor IL‐6 to fall below normal levels, thereby interfering with the normal phosphorylation of Radixin.

## AUTHOR CONTRIBUTIONS

Shengran Wang: Writing – original draft, Investigation and data collection. Sixuan Wang: Construction of animal model and investigation. Zhun Wang: Animal behavior testing and management. Jinpeng Dong: Animal feed and management. Mengxue Zhang: Image analysis. Yongan Wang: Methodology. Jianyu Wang: Result validation. Beichen Jia: Data collection. Yuan Luo: Funding supervision. Yiqing Yin: Project administration, Funding acquisition, Investigation and Writing – draft investigation.

## FUNDING INFORMATION

This work was supported by the Scientific Research Program of Tianjin Education Commission of China (Key Program, 2022ZD063), the Tianjin Scientific Research Start‐up Foundation for Talent Introduction (ZLWKJZZL06), and the Tianjin Key Medical Discipline (Specialty) Construction Project (TJYXZDXK‐009A). We would like to thank Yun Feng and Chunliu Liu from Center for Biological Imaging (CBI), Institute of Biophysics, Chinese Academy of Sciences for their help of analyzing confocal images.

## CONFLICT OF INTEREST STATEMENT

The author(s) declare that they have no potential conflicts of interest.

## Supporting information


Figure S1.

Figure S2.

Figure S3.

Figure S4.

Figure S5.

## Data Availability

The data that support the findings of this study are available from the corresponding author upon reasonable request.
