# Supplementary figures and images for: The changing of α5‐GABAA receptors expression and distribution participate in sevoflurane‐induced learning and memory impairment in young mice
Source: CNS Neurosci Ther. 2024 May 2;30(5):e14716. doi: 10.1111/cns.14716 (PMC11066188; doi:10.1111/cns.14716)

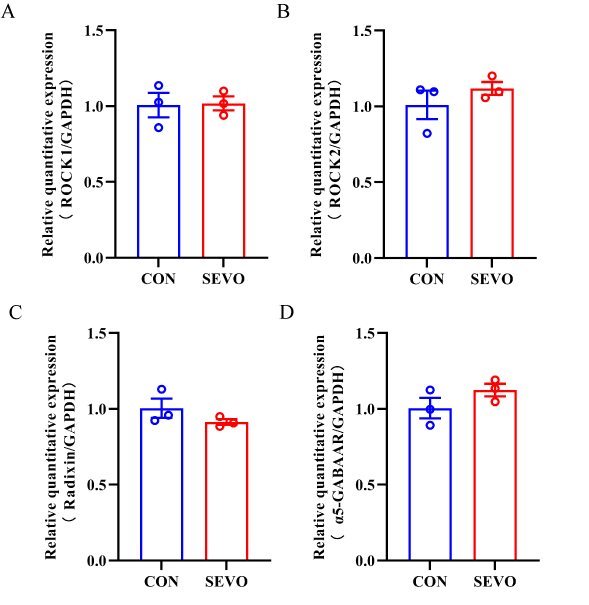

Supplement: Supplementary file 1 — Figure S1. [file CNS-30-e14716-s001.tif]

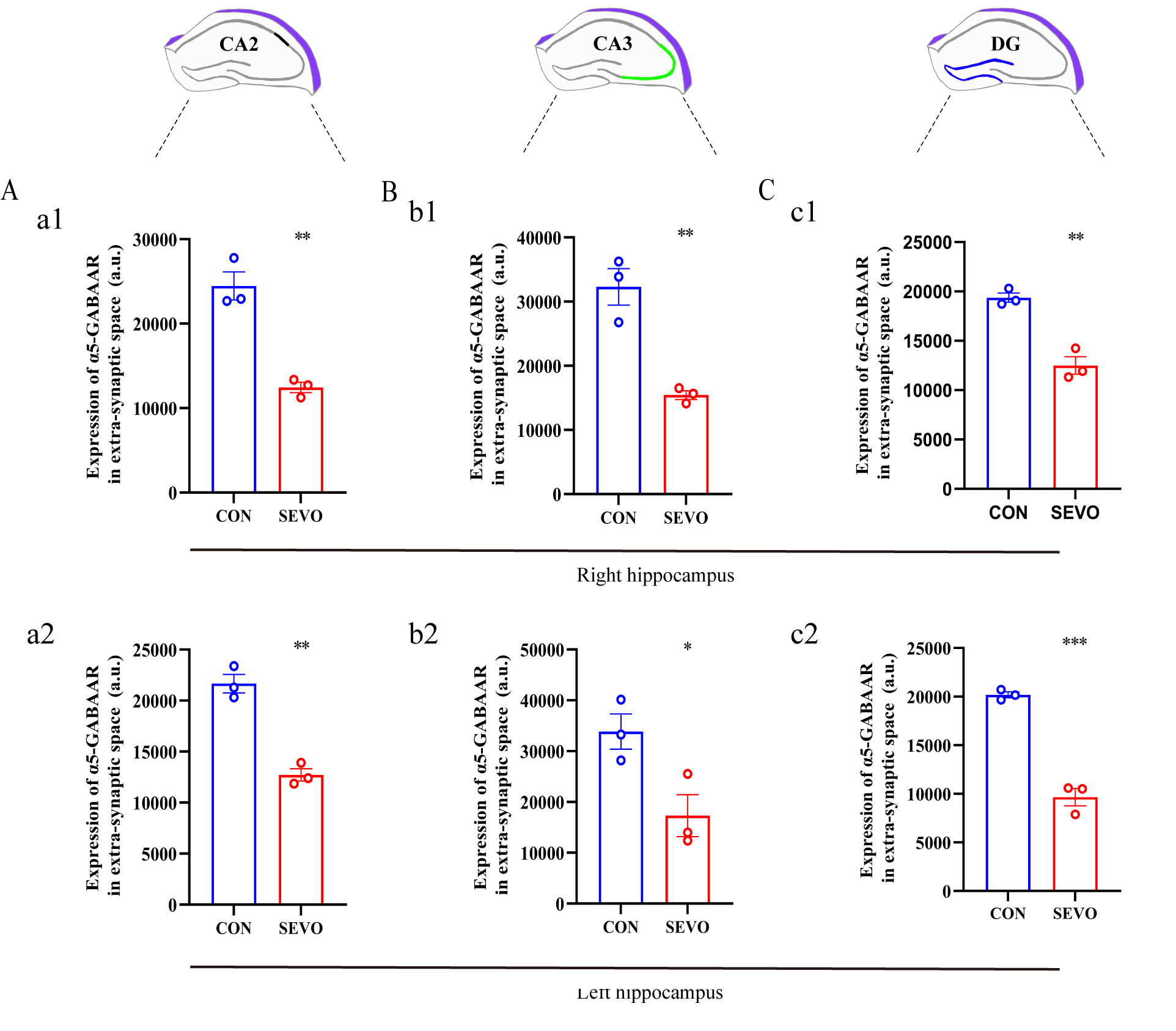

Supplement: Supplementary file 2 — Figure S2. [file CNS-30-e14716-s005.tif]

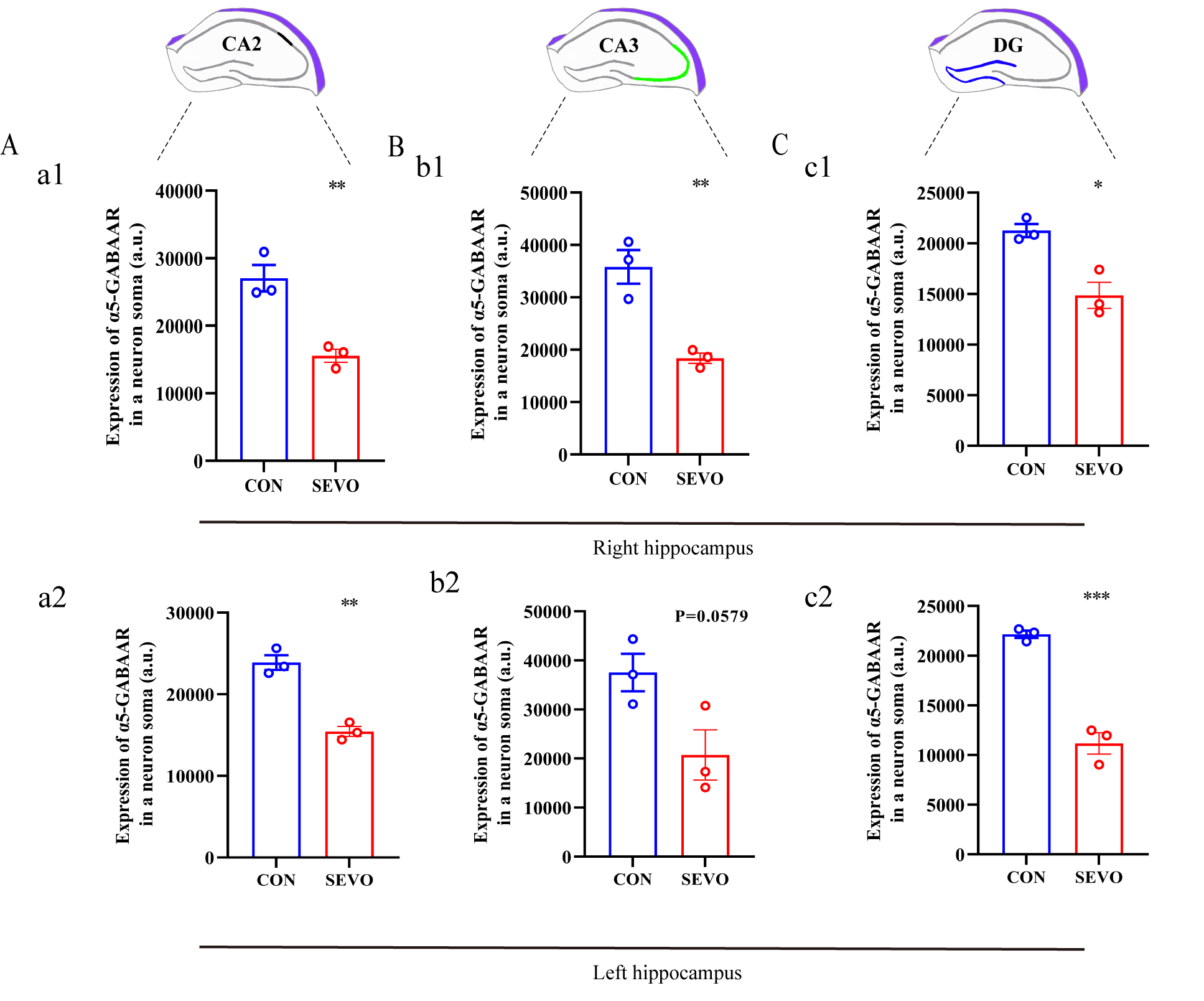

Supplement: Supplementary file 3 — Figure S3. [file CNS-30-e14716-s003.tif]

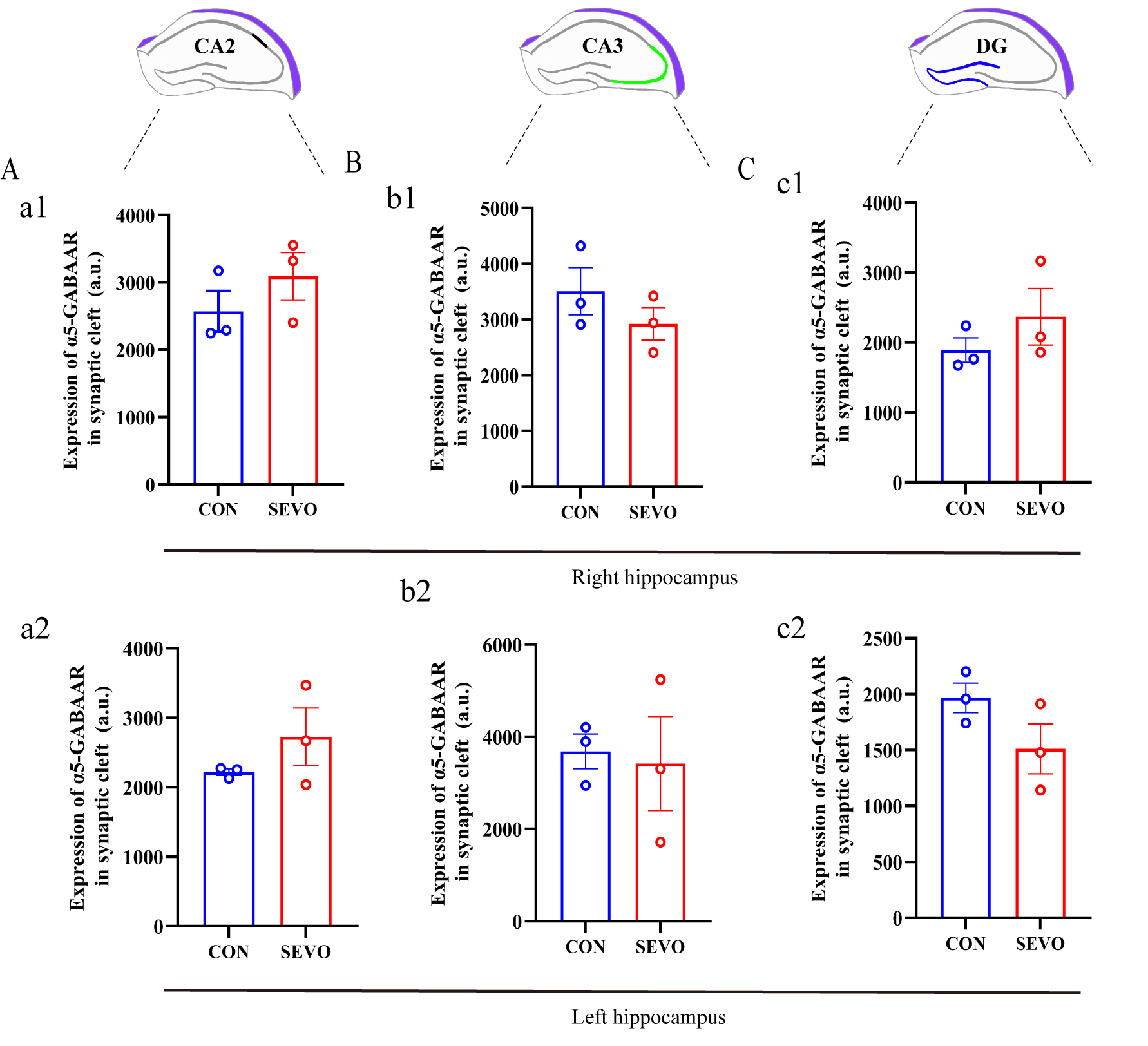

Supplement: Supplementary file 4 — Figure S4. [file CNS-30-e14716-s002.tif]

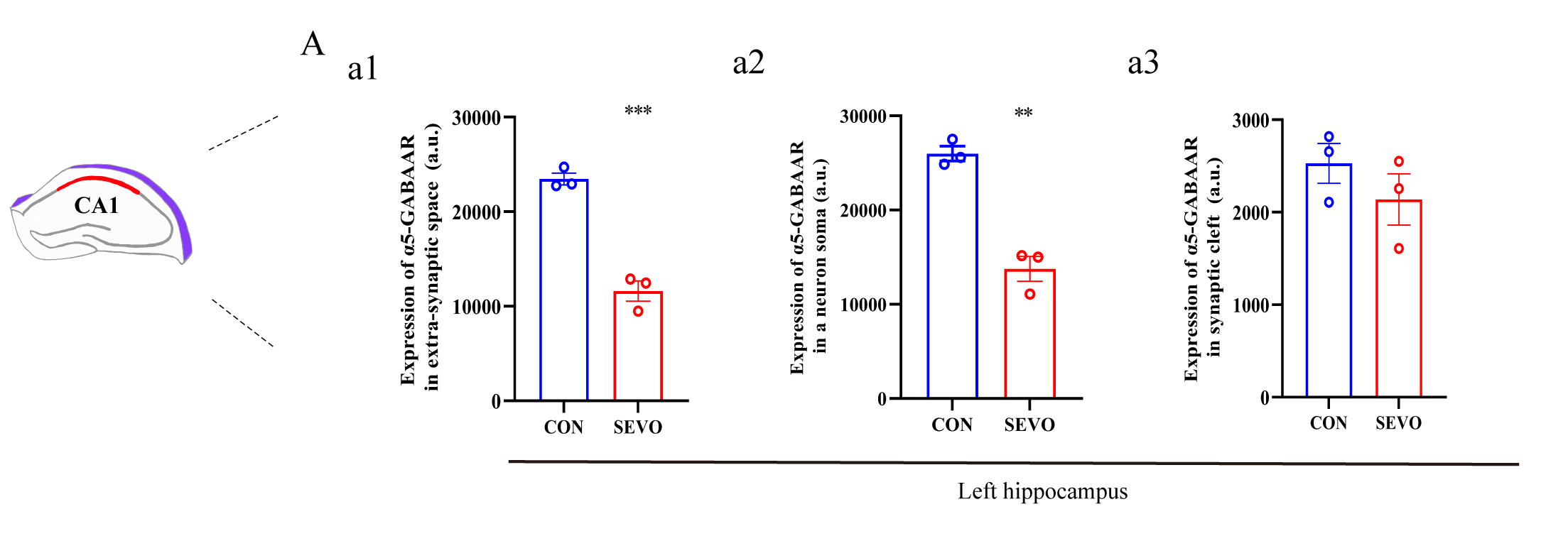

Supplement: Supplementary file 5 — Figure S5. [file CNS-30-e14716-s004.tif]
